# Elevated plasma neurofilament light and glial fibrillary acidic protein are associated with impaired cerebrovascular reactivity in older adults

**DOI:** 10.1002/alz.087697

**Published:** 2025-01-09

**Authors:** Arunima Kapoor, Shubir Dutt, Amy Nguyen, Aimée Gaubert, John Paul M Alitin, Isabel Sible, Anisa J Marshall, Fatemah Shenasa, Allison C Engstrom, Xingfeng Shao, Alessandra Cadete Martini, Elizabeth Head, Danny JJ Wang, Daniel A. Nation

**Affiliations:** ^1^ University of California, Irvine, Irvine, CA USA; ^2^ University of Southern California, Los Angeles, CA USA; ^3^ The UC Irvine Institute for Memory Impairments and Neurological Disorders (UCI MIND), Irvine, CA USA

## Abstract

**Background:**

Plasma neurofilament light (NfL) and glial fibrillary acidic protein (GFAP) are markers of axonal and astroglial injury, respectively. Both markers have been proposed as predictive biomarkers of cerebral small vessel disease, with elevated levels indicating higher burden of white matter hyperintensities, lacunar infarcts and cerebral microbleeds. However, to date, no study has examined whether NfL and GFAP levels are associated with dynamic markers of small vessel damage such as cerebrovascular reactivity (CVR)—the ability of cerebral blood vessels to regulate cerebral blood flow (CBF) in response to vasodilatory or vasoconstrictive stimuli. In this study, we examined whether levels of NfL and GFAP are associated with CVR to hypercapnia and hypocapnia.

**Method:**

Independently living older adults (N = 78; mean age = 69.5 years; SD = 8.1; age range 55‐89 years; 35.9% male) free of dementia or clinical stroke were recruited from the community and underwent venipuncture and brain MRI. Plasma levels of NfL, and GFAP were determined using a digital immunoassay. Pseudo‐continuous arterial spin labeling MRI quantified whole brain cerebral perfusion during CVR to hypocapnia (0.1Hz paced breathing; vasoconstriction) and hypercapnia (15s breath holds, vasodilation).

**Result:**

A significant negative association was observed between circulating levels of NfL and global CVR to hypocapnia (r = ‐.26, p = .027) and hypercapnia (r = ‐.23, p = 0.049) as well as GFAP and CVR to hypercapnia (‐.26, p = .025).

**Conclusion:**

This study demonstrates that elevated levels of NfL and GFAP are associated with diminished CVR to hypercapnia and levels of NfL are associated with impaired CVR to hypocapnia. These findings are consistent with prior studies suggesting that plasma NfL and GFAP may serve as biomarkers of cerebral small vessel disease. Given that these markers signify axonal and astroglial injury, these findings also provide insight into the pathophysiological processes underlying cerebrovascular dysfunction, which may contribute to the development of neurodegenerative conditions.

